# Evaluation of a Diagnostic and Management Algorithm for Adult Caustic Ingestion: New Concept of Severity Stratification and Patient Categorization

**DOI:** 10.3390/jpm12060989

**Published:** 2022-06-17

**Authors:** Yu-Jhou Chen, Chen-June Seak, Hao-Tsai Cheng, Chien-Cheng Chen, Tsung-Hsing Chen, Chang-Mu Sung, Chip-Jin Ng, Shih-Ching Kang, Ming-Yao Su, Sen-Yung Hsieh

**Affiliations:** 1 Department of Gastroenterology and Hepatology, Linkou Chang Gung Memorial Hospital, Taoyuan 333, Taiwan; chenyujhou@gmail.com (Y.-J.C.); q122583@adm.cgmh.org.tw (T.-H.C.); song3329@cgmh.org.tw (C.-M.S.); siming@adm.cgmh.org.tw (S.-Y.H.); 2 Division of Gastroenterology and Hepatology, Department of Internal Medicine, New Taipei Municipal TuCheng Hospital; No.6, Sec2, Jincheng Road, Tucheng Dist., New Taipei City 236, Taiwan; doctorsu@adm.cgmh.org.tw; 3 Department of Emergency Medicine, New Taipei Municipal TuCheng Hospital, New Taipei City 236, Taiwan; julianseak@hotmail.com; 4 Department of Emergency Medicine, Linkou Chang Gung Memorial Hospital, Taoyuan 333, Taiwan; ngowl@ms3.hinet.net; 5 School of Medicine, College of Medicine, Chang Gung University, Taoyuan 333, Taiwan; chenchiencheng@cgmh.org.tw; 6 Graduate Institute of Clinical Medicine, College of Medicine, Chang Gung University, Taoyuan 333, Taiwan; 7 Department of Medical Image and Intervention, Linkou Chang Gung Memorial Hospital, Taoyuan 333, Taiwan; 8 Division of Trauma and Emergent Surgery, Linkou Chang Gung Memorial Hospital, Taoyuan 333, Taiwan; sckang1011@gmail.com; 9 Taiwan Association for the Study of Small intestinal Disease (TASSID), Taoyuan 333, Taiwan

**Keywords:** caustic ingestion, corrosive injury, algorithm, flowchart, endoscopy, esophagogastroduodenoscopy, computed tomography, surgery, severity stratification, patient categorization

## Abstract

Background: Caustic ingestion has gained increasing attention worldwide. However, the insight into whether to use esophagogastroduodenoscopy (EGD) or computed tomography (CT) for first-line investigation remains controversial. This study aimed to evaluate a diagnostic and management algorithm that combines EGD and CT for rapid triage. Methods: We established an algorithm for our hospital in 2013, aiming to maximize the benefits and minimize the limitations of EGD and CT. Then, we retrospectively analyzed the 163 enrolled patients treated between 2014 and 2019 and categorized them into 4 groups: A = 3 (1.8%): with perforation signs and directly confirmed by CT, B = 10 (6.1%): clinically suspected perforation but not initially proven by CT, C = 91 (55.8%): initial perforation less favored but with EGD grade ≥ 2b or GI/systemic complications, and D = 59 (36.2%): clinically stable with EGD grade ≤ 2a, according to initial signs/symptoms and EGD/CT grading. The morbidity and mortality of each group were analyzed. The predictive values of EGD and CT were examined by logistic regression analyses and receiver operating characteristic (ROC) curves. Results: The outcomes of such algorithm were reported. CT was imperative for patients with toxic signs and suspected perforation. For non-emergent operations, additional EGD was safe and helpful in identifying surgical necessity. For patients with an initially low perforation risk, EGD alone sufficiently determined admission necessity. Among inpatients, EGD provided excellent discrimination for predicting the risk for signs/symptoms’ deterioration. Routine additional CT was only beneficial for those with deteriorating signs/symptoms. Conclusions: According to the analyses, initial signs/symptoms help to choose EGD or CT as the first-line investigative tool in caustic patients. CT is necessary for seriously injured patients, but it cannot replace EGD for moderate/mild injuries. The severity stratification and patient categorization help to simplify complex scenarios, accelerate decision-making, and prevent unnecessary intervention/therapy. External validation in a larger sample size is further indicated for this algorithm.

## 1. Introduction

With the increasing attention received worldwide, caustic ingestion has remained complex because of the wide-ranging gastrointestinal (GI) tract damage and the miscellaneous complications. Pathophysiologically, strong acids form coagulative necrosis/eschar, which limit deepening, whereas strong alkalis present saponification/liquefaction effects and cause penetration [1,2,3,4,5]. Risks of mortality and perforation seem dose-dependent for acids and pH-dependent for alkalis [6]. Furthermore, age, sex, psychiatric/systemic comorbidities, and socioeconomic status play important roles in survival prognosis and post-injury quality of life [1,2,3,4,5,7,8,9,10].

Given the complexity of caustic GI tract injury, evidence-based guidelines for early survey and management are imperative. Numerous studies thoroughly discussed the pros and cons of various protocols, especially regarding esophagogastroduodenoscopy (EGD) or computed tomography (CT) as the first-line investigative tool [1,3,6,10,11,12,13,14,15,16,17,18,19,20,21,22]. However, most studies were merely debating on choosing either EGD or CT for all caustic ingestion scenarios, despite the different limitations of such tools for specific situations. Presently, scarce algorithms can practically and effectively combine these tools for rapid triage.

Hence, we tried to propose a new practical flowchart that rapidly categorizes patients and accordingly choose either EGD or CT for first-line investigation. This study aimed to evaluate this algorithm that combines EGD and CT for rapid triage.

## 2. Materials and Methods

### 2.1. Algorithm Establishment and Study Population

**Figure 1:**
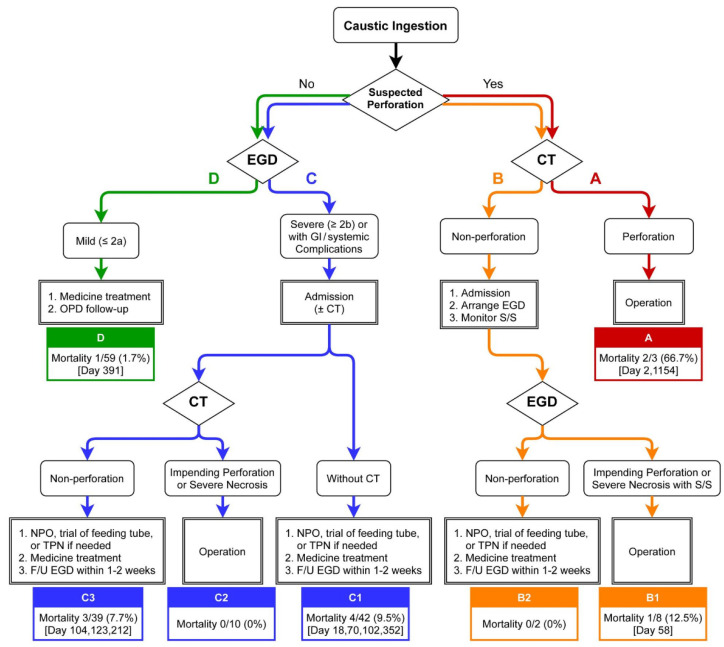
Reported flowchart of patient categorization and management for caustic ingestion used in Linkou Chang Gung Memorial Hospital (CGMH). Abbreviations: CT, computed tomography; EGD, esophagogastroduodenoscopy; F/U, follow-up; GI, gastrointestinal; NPO, nulla per os (nothing by mouth); OPD, out-patient department; S/S, signs/symptoms; TPN, total parenteral nutrition.

**Figure 2:**
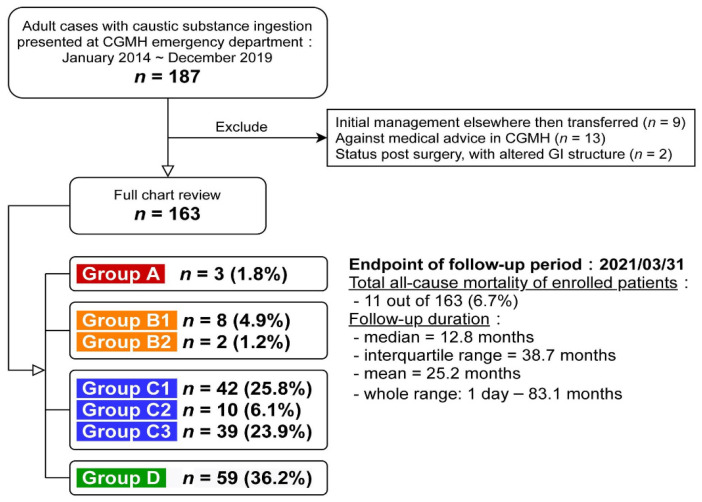
Enrollment diagram of the study population. Abbreviations: CGMH, Chang Gung Memorial Hospital; GI, gastrointestinal.

In 2013, colleagues from the Department of Gastroenterology and Hepatology, Emergency Medicine, Medical Image and Intervention, and Trauma and Emergent Surgery in Linkou Chang Gung Memorial Hospital (CGMH), the largest referral center in Taiwan, established an algorithm for the initial management of adult caustic ingestion for first-line physicians in our hospital. All patients received multidisciplinary care. Of note, the exact clinical decisions and whether to totally adopt this algorithm still depend on each physician. Later, we retrospectively identified 187 adults (≥18 years old) treated for caustic ingestion between January 2014 and December 2019 at Linkou CGMH. Most patients received management that was consistent with our algorithm and was further categorized as the reported flowchart (Figure 1), and 24 were excluded (9 already received initial management elsewhere, 13 were against medical advice, and 2 had an altered GI tract structure after previous surgery). For the 163 enrolled patients (Figure 2), a full chart review was conducted to identify the clinical courses and categorize them by group (groups A, B, C, and D). The CGMH Institutional Review Board approved this study (202000583B3).

### 2.2. Patient Categorization

The reported flowchart (Figure 1) combines EGD and CT for triage and categorizes the patients into groups A, B (B1/B2), C (C1/C2/C3), and D. The decision-making processes were conducted by emergency physicians with multidisciplinary discussion. Gastroenterologists were consulted in all cases. General surgeons co-evaluated the operative indication in groups A/B/C according to criteria as follows.

For patients initially found with peritoneal signs or extreme abdominal/chest pain, with or without unstable vital signs, hollow-organ perforation is highly suspected; thus, urgent CT is immediately required. Once perforation is confirmed, surgery should be performed early (group A). If CT preliminarily excludes perforation, EGD is further performed to stratify the severity of the GI tract injury. Deterioration of signs/symptoms suggests progression to severe necrosis and/or even delayed perforation, making operation inevitable in some patients (group B1). The signs of deterioration contain three components, as follows. First, unstable vital signs mainly included shock, either septic or hemorrhagic. Second, delayed observed peritoneal sign, which was not initially detected at the first triage. Third, extreme abdominal/chest pain that could not be explained by other clinical etiology and possibly could not even be relieved by weak-opioid pain control. The combination of EGD grading and close monitoring of clinical status prevents other patients from unnecessary operation (group B2) who simply recover from conservative treatment. For the patients with drowsy or stupor mentality which prevent clinicians from quick physical examination triage, an advanced image (CT scan) would be arranged because we could not immediately exclude that severe caustic injury (possibly with perforation) leads to systemic complications, including such conscious disturbance.

For patients with an initially low perforation risk, EGD is performed to determine admission necessity. Those with caustic injury ≥ Zargar’s 2b or GI/systemic complications require admission. Additional CT is not routinely arranged (group C1) because of the absence of documented evidence in Taiwan. In this study, the decision for additional CT was decided by the emergency medicine specialists and is discussed in the following sections. Patients with delayed impending perforation or severe necrosis would receive operation (group C2), whereas those with stable conditions would undergo conservative treatment (group C3). Meanwhile, patients with low-grade injury (≤Zargar’s 2a) and no other complications do not require admission, and they are prescribed with oral antacids and scheduled for outpatient clinic follow-up (group D).

### 2.3. EGD Survey

According to the algorithm, urgent EGD was arranged within 24 h after caustic ingestion. At CGMH, EGD is available around the clock. Experienced endoscopists performed urgent EGDs using standard upper GI endoscopes (GIF XQ-230 (9.2 mm), GIF Q-240X (9.4 mm), GIF Q-260J (9.9 mm), and GIF Q-260 (9.2 mm); Olympus, Tokyo, Japan). Oral xylocaine spray was used, except in patients who required ventilation support under general anesthesia for respiratory difficulty or unclear consciousness. In severely injured patients, insufflation and retrovision maneuvers were carefully performed or avoided.

Caustic damage was graded using Zargar’s endoscopic classification (grades 0, 1, 2a, 2b, 3a, and 3b) [12]. A single endoscopist (the corresponding author) performed nearly half of the urgent EGDs. This endoscopist also reviewed the endoscopic photos of all other enrolled patients and confirmed the Zargar’s grades for consistency and accuracy. We excluded 6 grades in the duodenum and 1 in the stomach for incomplete EGD studies resulting from patient intolerance.

### 2.4. CT Survey

According to the algorithm, urgent CT was arranged within 24 h after caustic ingestion. At CGMH, CT is available around the clock. An experienced radiologist confirmed all injury grades under enhanced thoracoabdominal CT (multidetector scanner; LightSpeed QX/i Scanner (Montreal, QC, Canada), General Electric Medical Systems, Milwaukee, WI, USA) for the esophagus, stomach, and duodenum. The protocol was to use intravenous contrast media, but patients with kidney insufficiency were excluded. Since our algorithm was established by 2013, we adopted the severity classification system (grades 1, 2, 3, and 4) proposed in 2010 [14], which was also advocated by the latest cornerstone review article [1].

### 2.5. Post-Caustic Ingestion Patient Care

Baseline hematological and biochemical laboratory data acquired during admission were collected. We reviewed the personal information, intent/substance/amount of ingestion, psychiatric/systemic comorbidities, treatment courses, GI/systemic complications, and survival outcomes of each patient. Substances with pH < 2 or > 12 were considered strong caustics. Psychiatric comorbidities were diagnosed/confirmed by psychiatrists through face-to-face interviews, based on the Diagnostic and Statistical Manual of Mental Disorders Fifth Edition (DSM-5).

Patients admitted to the gastroenterology department were conservatively treated with proton pump inhibitors or H_2_ antagonists for caustic injury, followed by parenteral nutrition without oral intake until clinically stable. If infection was suspected, blood cultures were obtained before administering antibiotics. When destabilization or respiratory difficulty occurred, the patient was transferred to the intensive care unit (ICU). The corticosteroid therapy was not prophylactically administered for preventing caustic strictures due to the limited overall quality of the evidence. Psychiatrists were consulted for face-to-face interviews and further therapy planning for suicidal ingestion cases and those already known to have underlying psychiatric disorders. Other specialists participated in treating specific complications. Follow-up EGD was performed within 1–2 weeks, as indicated. When discharged, patients were followed up in the out-patient clinic for at least 6 months.

### 2.6. Complications

Any complications during follow-up were recorded [6,13]. Upper GI complications included bleeding, perforation, stricture formation, and delayed fistula formation. Melena/hematemesis/coffee-ground vomitus indicated bleeding. Perforation and fistula were diagnosed using CT or endoscopy. Dysphagia/regurgitation/odynophagia indicated stricture, as confirmed by endoscopy or upper GI radiography.

Systemic complications included aspiration injury, respiratory failure, hepatic injury, kidney injury, septic shock, and disseminated intravascular coagulation. Clinical signs/symptoms and scanned images were considered when diagnosing aspiration injury and/or respiratory failure. Aspartate transaminase (AST/GOT) or alanine aminotransferase (ALT/GPT) elevation to three times the normal upper limit indicated hepatic injury. Kidney injury was defined according to KDIGO 2012 clinical practice guidelines [23]. Sepsis associated with hypotension and perfusion abnormalities despite adequate fluid (volume) resuscitation indicated septic shock [24].

### 2.7. Statistical Analyses

We express continuous variables as the median and interquartile range (IQR = Q3 − Q1) or range, and categorical variables as numbers with percentages. When calculating percentages, we excluded patients with missing values from the chart review. All statistical tests were two-sided and performed using Statistical Product and Service Solutions version 26 (IBM, Armonk, NY, USA). The categorical variables were assessed via Pearson’s χ^2^ test or Fisher’s exact test. For nonparametric continuous variables, we used the Mann–Whitney U test. The association among covariates was explored by univariate and multivariate logistic regression analyses using the odds ratio (OR) and 95% confidence interval (CI). Diagnostic abilities are illustrated using the ROC curves with the area under the ROC curve (AUROC). Survival was evaluated by univariate analysis using the Kaplan–Meier method, and differences between survival curves were assessed via the log-rank test. The overall survival and all-cause mortality were determined, with the endpoint of the follow-up period in March 2021. A *p* < 0.05 was considered statistically significant.

## 3. Results

### 3.1. Patient Characteristics

The 163 enrolled patients comprised 76 males (46.6%) and 87 females (53.4%), and the median age at caustic ingestion was 50 years (IQR = 28; range, 18–100). Suicidal ingestion was reported in 126 patients (77.3%). The median ingested amount was 100 mL (IQR = 183; range, 5–1000). Additionally, 42.9% of ingested substances (47 strong acids, 23 strong alkalis) were strong caustic compounds. Three-fourths of the patients had psychiatric comorbidities.

The median follow-up was 12.8 months (IQR = 38.7; range, 1 day to 83.1 months). The overall survival rates by the Kaplan–Meier method at 3 and 6 months and 1, 2, 3, and 5 years were 97.3%, 94.9%, 92.9%, 91.7%, 91.7%, and 89.1%, respectively.

Figure 1 reveals the patient numbers with percentages and the survival condition of each group (A, *n* = 3, 1.8%; B, *n* = 10, 6.1%; C, *n* = 91, 55.8%; D, *n* = 59, 36.2%). EGD was performed in all cases in groups B/C/D (*n* = 160, 98.2%). All patients in groups A/B/C2/C3 and a few in group D received CT (*n* = 67, 41.1%). Patients in groups A/B1/C2 underwent surgical intervention (*n* = 21, 12.9%), which revealed six perforation cases (28.6%) and severe necrosis in some cases, as confirmed by pathologic reports. Table 1 shows the comprehensive demographic profiles of each group.

### 3.2. EGD Grading

**Figure 3:**
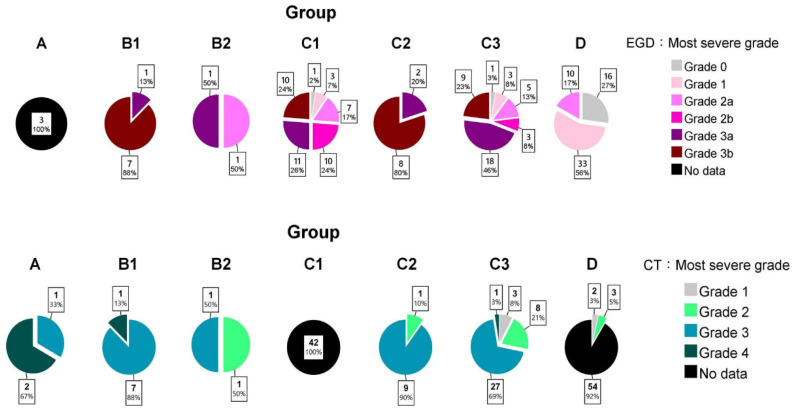
The most severe grade of each patient group estimated using EGD and CT.

Figure 3 presents the EGD grades of each group. The stomach obtained the highest grade in most cases, followed by the esophagus and duodenum. In group B, those requiring operation (B1) were significantly more severe (*p* = 0.044) than those treated conservatively (B2). In group C, the surgical group (C2) was also more severe (*p* < 0.001) than the conservative group (C1/C3).

### 3.3. CT Grading

Figure 3 shows the distribution of CT grades. Only four patient stomachs had grade 4 injuries, of which three underwent operation. Compared with esophageal and stomach injuries, duodenal damages were relatively trivial in most patients, including those who required operation. In a nonparametric setting, the CT grades were not significantly different between B1 and B2 (*p* = 0.356) and between C2 and C1/C3 (*p* = 0.358).

### 3.4. Survival Outcomes

**Figure 4:**
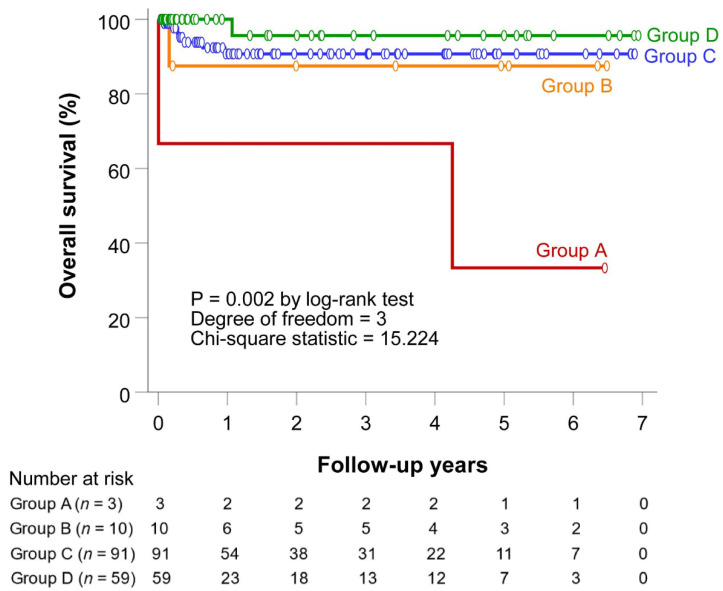
Overall survival of each patient group.

The overall survival in each group was illustrated using the Kaplan–Meier method (*p* = 0.002, Figure 4). The pairwise comparisons via log-rank tests of the overall survival were significant in A–C1 (*p* = 0.013), A–C2 (*p* = 0.034), and A–D (*p* < 0.001).

### 3.5. Roles of EGD/CT in Group C

Over half of the patients belonged to group C, and all of them received EGD. The characteristics, morbidities, and mortalities were compared between C1 (not receiving CT, *n* = 42) and C2/C3 (receiving additional CT, *n* = 49) (Table 2). The listed etiologic variables, EGD severity, systemic/GI complications, and overall survival showed no significance.

For those patients receiving both EGD and CT (C2/C3), the correlation between the EGD/CT grading systems and operation requirement was evaluated by logistic regression (Table 3). In univariate settings, EGD grade was statistically significant (OR = 8.556; 95% CI, 1.622–45.136; *p* = 0.011) in distinguishing patients with (C2) and without (C3) operation. Conversely, CT grade (OR = 2.250; 95% CI, 0.497–10.178; *p* = 0.292) and age (OR = 0.960; 95% CI, 0.919–1.003; *p* = 0.069) were not significant. In multivariate analysis, EGD grade remained significant (OR = 8.555; 95% CI, 1.559–46.942; *p* = 0.013).

**Figure 5:**
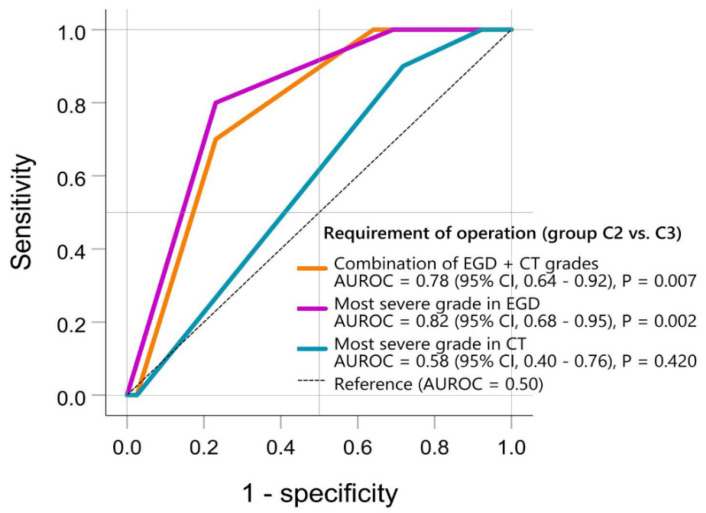
Grades of esophagogastroduodenoscopy (EGD) and computed tomography (CT) in differentiating group C2 (requiring operation) and group C3 (not requiring operation). The “most severe EGD/CT grades” refers to the most severe grade among the esophagus, stomach, and duodenum in each patient under EGD (Zargar’s 0, 1, 2a, 2b, 3a, 3b) and CT (grades 1, 2, 3, 4), respectively. The “combination of EGD + CT grades” is calculated by EGD (Zargar’s 0, 0 point; Zargar’s 1, 1 point; Zargar’s 2a, 2 points; Zargar’s 2b, 3 points; Zargar’s 3a, 4 points; Zargar’s 3b, 5 points) plus CT (grade 1, 1 point; grade 2, 2 points; grade 3, 3 points; grade 4, 4 points). Hence, the “combination of EGD + CT grades” ranges from 1 to 9 points. Abbreviations: AUROC, area under the receiver operating characteristic curve; CI, confidence interval; CT, computed tomography; EGD, esophagogastroduodenoscopy.

In C2/C3, the correlation between EGD/CT grades and operation requirement was further examined by plotting the ROC curve (Figure 5). The AUROC of EGD was 0.82 (95% CI, 0.68–0.95; *p* = 0.002), while that of CT was 0.58 (95% CI, 0.40–0.76; *p* = 0.420). The combination of EGD and CT grades had an AUROC = 0.78 (95% CI, 0.64–0.92; *p* = 0.007). The AUROC of EGD grade alone was not further increased by the additional CT grade.

## 4. Discussion

This study reports the algorithm in Linkou CGMH combining EGD and CT in managing adult caustic ingestion. By retrospectively examining the outcomes of each patient group, we also found a new concept of severity stratification to simultaneously maximize the benefits and minimize the limitations of EGD and CT. Patients with toxic signs and suspected perforation required urgent CT. For non-emergent operations, additional EGD seemed to be safe and helpful. In patients with an initially low perforation risk, EGD alone sufficiently determined admission necessity. Except for those with deteriorating signs/symptoms, additional CT brings no benefit in most cases.

In 2020, Hoffman et al. [1] thoroughly reviewed the cornerstone studies and schemed out a detailed algorithm for the diagnosis and management of caustic ingestions. However, physicians might hardly obtain the information of acid/alkaline property or intentional/unintentional motivation at initial assessment, and it is thereby difficult to directly initiate the evidence-based algorithm. Our algorithm offers physicians another way of categorizing patients. Primary grouping was achieved by prompt physical examination and clinical signs/symptoms, identifying whether perforation is suspected (group A/B or C/D). Although initial symptoms do not perfectly correlate with the extent of damage [2,4,5,25], signs/symptoms’ assessment did effectively categorize patients according to the aforementioned analyses in our reported experiences. Therefore, either EGD or CT could be a first-line investigative tool. For group A/B, CT is a key to secondary grouping (A or B) to determine the need for emergent operation or additional EGD. For group C/D, EGD alone is sufficient for determining admission necessity, and additional CT only benefits those with the following deteriorating signs/symptoms. This patient categorization helps to simplify the complicated scenarios, accelerate the decision-making, and prevent unnecessary intervention/therapy.

One-third of our patients belonged to group D. After being graded ≤ 2a via EGD and without complication, all of them did not need admission. The overall survival rate was near 100%, and none of them required re-admission throughout the follow-up period: only one patient died from cardiogenic etiology over one year later. Thus, a grade of ≤2a requires only simple medication and outpatient clinic follow-up and is thereby cost-effective in Taiwan.

Group C comprised half of the patients. All of them had severe GI tract damage (grade ≥ 2b) according to EGD triage, and/or GI/systemic complications. Given the lack of previous literature about additional CT in such patients, additional CT requirement depended on the discretion of emergency medicine specialists, with multidisciplinary discussion, in our hospital. Based on the analyses among those with stable signs/symptoms, routine additional CT did not improve the morbidities and mortalities in groups C2/C3. Nevertheless, CT remained essential for those with deteriorating signs/symptoms. In other words, patients in group C do not need routine CT, except for those with exacerbation and suspected surgery requirement. Under such algorithm, the reported mortality causes in group C were as follows: one postoperative aspiration pneumonia, one community-acquired pneumonia, one suicide by hanging, two terminal-staged malignancy, and two from acute coronary syndrome. Therefore, not routinely performing urgent CT did not lead to missed operation. Certainly, the decision of surgery in clinical practice could not be solely based on the endoscopic examination findings. For those that received operations due to deteriorating signs/symptoms, combination of CT and EGD findings additionally provided surgeons with the length and margin of necrosis while deciding the resection border.

Meanwhile, the EGD grading system seemed more powerful in evaluating the operation requirement in group C. Zargar’s severity classification had a statistical correlation with surgical necessity in both univariate and multivariate settings. The AUROC of EGD grading was 0.82, which indicates excellent discrimination [26]. However, the CT grade demonstrated no statistical significance (AUROC = 0.58, poor discrimination [26]) in differentiating C2 and C3 in our study. Of note, the combination of EGD and CT grading showed acceptable discrimination [26] (AUROC = 0.78), but it did not further add predictive value to the EGD-originated AUROC. Such results are also illustrated in Figure 3. Severe injuries confirmed by EGD (especially Zargar’s 3b) had a high risk for signs/symptoms’ deterioration (C2), but most C2/C3 patients had CT grade 3, with no difference between such groups. Based on such results, EGD alone is suggested to be sufficient in determining operation necessity in group C. This conclusion seems to be contrary to those studies advocating emergency decision-making by CT alone (focusing on high-grade necrosis) [17,18]. However, this discordance could be explained by the difference in the severity of the selected patients. CT overcomes the limitation of EGD through its advantages for those seriously injured, in line with our group A/B cases. However, when mixed with the mildly/moderately injured cases (in line with our group C/D), statistical significance might not be found. Hence, Bonnici et al. [22] reviewed numerous studies and concluded that CT use in mixed circumstances is insufficient to totally replace endoscopy as the first-line investigative tool. Consequently, the importance of initial severity stratification, patient categorization, and a comprehensive decision-making process is further highlighted.

Only few patients were diagnosed with perforation through urgent CT (group A) at the initial assessment at the emergency department. Similar to other physical trauma cases, serious injuries had occurred before arrival at the emergency department. Clinicians could only be committed to early diagnosis and surgical management to limit the extent of morbidity and mortality, which had been well-discussed and documented in the past decade. In such scenarios, CT is imperative, and EGD would be redundant. In other cases, however, CT preliminarily excludes perforation (group B). The caustic damage (especially CT grades 3 and 4) could have a chance to recover conservatively but also might still progress during the following hours. Moreover, the roles of CT/EGD for operation have long been controversial [2,3]. In this study, additional urgent EGD was carefully performed. The severity under EGD (*p* = 0.044), not CT (*p* = 0.356), significantly correlated with operation requirement. Additional EGD grading seemed to better predict the risk for signs/symptoms’ deterioration in our retrospective analyses. Regarding EGD safety, no patient had perforation complications. According to final pathology reports, three operated cases had gastric perforation (deteriorating signs/symptoms noted 5, 6, and 8 h after EGD in these three patients); considering the timeframe, direct intra-EGD perforation could be excluded. The five other operated cases in group B had focal to extensive transmural necrosis (deteriorating signs/symptoms noted 1, 3, 4, 4, and 17 h after EGD). In consideration of preserving the native GI tract, our protocol prevented two group B2 patients from unnecessary esophagectomy/gastrectomy. To summarize, CT excluded initial perforation, and EGD safely provided predictive insights for surgical necessity, indication, and information for the resection border. The combination of CT and EGD in group B patients was beneficial, with more precise surgical indication.

Our study has limitations. First, it is retrospective in nature and has a small sample size. Memory lapses and clerical errors might have led to information bias while chart recording. Nevertheless, the cross-validation and double-check processes during the full chart review may have reduced this inevitable bias. Second, all caustics may not be alike. Caustic ingestion etiologies exhibit geographic and cultural differences. Caustic substances also vary in commercial formula. Third, we reported the clinical practice under National Health Insurance in Taiwan. In other nations/regions, the accessibility of medical resources and cost-effective balance may differ; for example, several factors might delay/hinder endoscopy [21,27]. If so, applications of this algorithm require modification, but the stratified concept of the EGD/CT combination remains helpful. Fourth, patients with lesions ≥ Zargar 2b were not treated with corticosteroids and the analyzed outcome was mainly focused on survival. Finally, the patient selection strategies for those previous studies [2,11,17], which concluded CT alone as the preferred investigative tool, were different from the composition in our group C. Therefore, the results were unable to be directly compared. To comprehensively validate this algorithm, future studies based on external populations are necessary.

## 5. Conclusions

We reported our algorithm for adult caustic ingestion to maximize the benefits and minimize the limitations of EGD and CT. According to the retrospective analyses, it seems reliable using initial signs/symptoms to choose either EGD or CT as the first-line investigative tool. CT plays paramount roles for seriously injured patients, but it cannot replace EGD for moderate/mild injuries. Such severity stratification and patient categorization help to simplify complex scenarios, accelerate decision-making, and prevent unnecessary intervention/therapy. Future studies based on external populations are essential to comprehensively validate this algorithm.

## Figures and Tables

**Table 1:** Comprehensive demographic profiles of each group of patients.

Variable	Group A	Group B1	Group B2	Group C1	Group C2	Group C3	Group D	Total
	(*n* = 3, 1.8%)	(*n* = 8, 4.9%)	(*n* = 2, 1.2%)	(*n* = 42, 25.8%)	(*n* = 10, 6.1%)	(*n* = 39, 23.9%)	(*n* = 59, 36.2%)	(*n* = 163, 100%)
Female	2 (66.7)	5 (62.5)	1 (50.0)	22 (52.4)	7 (70.0)	25 (64.1)	25 (42.4)	87 (53.4)
Age, year	64 (nil)	51 (37)	55 (nil)	48 (22)	45 (13)	55 (26)	43 (28)	50 (28)
Old (age ≥ 65)	1 (33.3)	3 (37.5)	1 (50.0)	8 (19.0)	1 (10.0)	15 (38.5)	14 (23.7)	43 (26.4)
Caustics substances								
Acid	3 (100)	4 (50.0)	1 (50.0)	19 (45.2)	6 (60.0)	23 (59.0)	25 (42.4)	81 (49.7)
Alkaline	0 (0)	4 (50.0)	1 (50.0)	19 (45.2)	4 (40.0)	13 (33.3)	25 (42.4)	66 (40.5)
Uncertain ^1^	0 (0)	0 (0)	0 (0)	4 (9.5)	0 (0)	3 (7.7)	9 (15.3)	16 (9.8)
Strong caustics (pH < 2 or >12)	3 (100)	6 (75.0)	1 (50.0)	20 (57.1)	5 (50.0)	21 (63.6)	14 (26.4)	70 (42.9)
Strong acids (pH < 2)	3 (100)	4 (50.0)	1 (50.0)	11 (26.8)	4 (40.0)	15 (44.1)	9 (15.8)	47 (28.8)
Strong alkalis (pH > 12)	0 (0)	2 (25.0)	0 (0)	9 (22.0)	1 (10.0)	6 (15.4)	5 (8.8)	23 (14.1)
Ingested amount, mL	150 (nil)	250 (200)	505 (nil)	100 (175)	50 (125)	100 (200)	50 (120)	100 (183)
Amount ≥ 100 mL	2 (66.7)	5 (83.3)	1 (50.0)	17 (58.6)	3 (42.9)	17 (63.0)	15 (34.1)	60 (50.8)
Suicidal ingestion	3 (100)	8 (100)	2 (100)	32 (76.2)	9 (90.0)	34 (87.2)	38 (64.4)	126 (77.3)
EGD grades								
Esophagus ≥ 2b	nil	7 (87.5)	1 (50)	21 (50.0)	9 (90.0)	17 (43.6)	0 (0)	55 (34.4)
Stomach ≥ 2b	nil	8 (100)	1 (50)	23 (54.8)	10 (100)	29 (76.3)	0 (0)	71 (44.4)
Duodenum ≥ 2b	nil	5 (62.5)	1 (50)	3 (7.7)	5 (50.0)	6 (16.7)	0 (0)	20 (12.5)
Incomplete EGD study	nil	0 (0)	0 (0)	3 (7.1)	0 (0)	3 (7.7)	0 (0)	6 (3.8)
ETT + MV during EGD	nil	5 (62.5)	0 (0)	8 (19.0)	3 (30.0)	8 (20.5)	0 (0)	24 (15.0)
CT grades								
Esophagus ≥ 3	3 (100)	5 (62.5)	0 (0)	nil	8 (80.0)	20 (51.3)	0 (0)	36 (53.7)
Stomach ≥ 3	3 (100)	8 (100)	1 (50.0)	nil	9 (90.0)	25 (64.1)	0 (0)	46 (68.7)
Duodenum ≥ 3	0 (0)	3 (37.5)	0 (0)	nil	0 (0)	2 (5.1)	0 (0)	5 (7.5)
Admission	3 (100)	8 (100)	2 (100)	42 (100)	10 (100)	39 (100)	0 (100)	104 (63.8)
Inpatient days	16 (nil)	31 (29)	8 (nil)	10 (10)	16 (13)	13 (18)	0 (0)	8 (16)
ICU admission	3 (100)	7 (87.5)	0 (0)	5 (11.9)	7 (70)	9 (23.1)	0 (0)	31 (19.0)
In-hospital mortality	1 (33.3)	1 (12.5)	0 (0)	1 (2.4)	0 (0)	0 (0)	0 (0)	3 (2.9)
Operation	3 (100)	8 (100)	0 (0)	0 (0)	10 (100)	0 (0)	0 (0)	21 (12.9)
Systemic complications	3 (100)	4 (50.0)	0 (0)	18 (42.9)	6 (60.0)	9 (23.1)	0 (0)	43 (26.4)
Aspiration injury	2 (66.7)	2 (25.0)	0 (0)	11 (26.2)	2 (20.0)	6 (15.4)	0 (0)	22 (13.5)
Respiratory failure	2 (66.7)	2 (25.0)	0 (0)	8 (19.0)	1 (10.0)	6 (15.4)	0 (0)	19 (11.7)
Hepatic injury	1 (33.3)	2 (25.0)	0 (0)	6 (14.3)	3 (30.0)	2 (5.1)	0 (0)	14 (8.6)
Renal injury	0 (0)	1 (12.5)	0 (0)	7 (16.7)	2 (20.0)	2 (5.1)	0 (0)	12 (7.4)
Septic shock	1 (33.3)	1 (12.5)	0 (0)	1 (2.4)	0 (0)	0 (0)	0 (0)	3 (1.8)
DIC	0 (0)	0 (0)	0 (0)	0 (0)	1 (10.0)	0 (0)	0 (0)	1 (0.6)
GI complications	3 (100)	5 (62.5)	1 (50.0)	16 (38.1)	5 (50.0)	16 (41.0)	0 (0)	49 (30.1)
Bleeding	3 (100)	4 (50.0)	1 (50.0)	8 (19.0)	2 (20.0)	7 (17.9)	0 (0)	25 (15.3)
Perforation	3 (100)	3 (37.5)	0 (0)	0 (0)	0 (0)	0 (0)	0 (0)	6 (3.7)
Stricture formation	1 (33.3)	1 (12.5)	0 (0)	9 (21.4)	2 (20.0)	13 (33.3)	0 (0)	0 (0)
Delayed fistula formation	0 (0)	0 (0)	0 (0)	0 (0)	1 (10.0)	0 (0)	0 (0)	1 (0.6)
Follow-up duration, months	50.9 (nil)	32.5 (69.8)	31.2 (nil)	13.0 (50.2)	33.8 (34.0)	15.4 (32.4)	6.1 (26.3)	12.8 (39.3)
All-cause mortality	2 (66.7)	1 (12.5)	0 (0)	4 (9.5)	0 (0)	3 (7.7)	1 (1.7)	11 (6.7)
Endoscopic dilation for stricture or obstruction	1 (33.3)	0 (0)	0 (0)	7 (16.7)	1 (10.0)	6 (15.4)	0 (0)	15 (9.2)
Operation for stricture or obstruction	0 (0)	1 (12.5)	0 (0)	3 (7.1)	0 (0)	6 (15.4)	0 (0)	10 (6.1)
Psychiatric comorbidities	3 (100)	7 (87.5)	2 (100)	35 (83.3)	9 (90.0)	33 (84.6)	37 (62.7)	126 (77.3)
Systemic comorbidities								
Hypertension	1 (33.3)	3 (37.5)	0 (0)	13 (31.0)	0 (0)	13 (33.3)	11 (18.6)	41 (25.2)
Diabetes mellitus	0 (0)	0 (0)	0 (0)	7 (16.7)	2 (20.0)	10 (25.6)	2 (3.4)	21 (12.9)
Cancer	0 (0)	1 (12.5)	0 (0)	2 (4.8)	1 (10.0)	5 (12.8)	1 (1.7)	10 (6.1)
Coronary artery disease	0 (0)	0 (0)	0 (0)	1 (2.4)	1 (10.0)	2 (5.1)	2 (3.4)	6 (3.7)

The data of continuous variables were expressed as the median and interquartile range (IQR = Q3 − Q1), and those of categorical variables were presented as numbers with percentages. Abbreviations: CT, computed tomography; DIC, disseminated intravascular coagulation; EGD, esophagogastroduodenoscopy; ETT + MV, endotracheal tube with mechanical ventilation; GI, gastrointestinal; ICU, intensive care unit. ^1^ The exact pH of ingested substances was uncertain in 16 (9.8%) patients at triage, and they received initial management following our algorithm. They were thus still included to prevent selection bias.

**Table 2:** Demographic characteristics between group C1 (not receiving CT) and group C2/C3 (receiving CT).

Variable	Group C1 (*n* = 42)	Group C2/C3 (*n* = 49)	*p*-Value
Female	22 (52.4)	32 (65.3)	0.285
Age, year	48 (22)	54 (26)	0.188
Strong caustics (pH < 2 or > 12)	20 (57.1)	26 (60.5)	0.82
Acid/Alkaline/Uncertain	19 (45.2)/19 (45.2)/4 (9.5)	29 (59.2)/17 (34.7)/3 (6.1)	0.405
Ingested amount ≥ 100 mL	17 (58.6)	20 (58.8)	>0.999
Suicidal ingestion	32 (76.2)	43 (87.8)	0.175
Psychiatric comorbidity	35 (83.3)	42 (85.7)	0.827
EGD grades			0.122
0/1	1 (2.4)/3 (7.1)	1 (2.0)/3 (6.1)	
2a/2b	7 (16.7)/10 (23.8)	5 (10.2)/3 (6.1)	
3a/3b	11 (26.2)/10 (23.8)	20 (40.8)/17 (34.7)	
ETT + MV during EGD	8 (19.0)	11 (22.4)	0.798
Inpatient days	10 (10)	14 (16)	0.297
In-hospital mortality	1 (2.4)	0 (0)	0.466
Overall survival ^1^	38 (90.5)	46 (93.9)	0.545
Systemic complications	18 (42.9)	15 (30.6)	0.276
Aspiration injury	11 (26.2)	8 (16.3)	0.305
Respiratory failure	8 (19.0)	7 (14.3)	0.582
Hepatic injury	6 (14.3)	5 (10.2)	0.749
Renal injury	7 (16.7)	4 (8.2)	0.334
Septic shock	1 (2.4)	0 (0)	0.462
DIC	0 (0)	1 (2.0)	>0.999
GI complications	16 (38.1)	21 (42.9)	0.674
Bleeding	8 (19.0)	9 (18.4)	>0.999
Perforation	0 (0)	0 (0)	–
Stricture formation	9 (21.4)	15 (30.6)	0.35
Endoscopic dilation for stricture	7 (16.7)	7 (14.3)	0.779

Continuous variables are expressed as the median and interquartile range (IQR = Q3 – Q1), and categorical variables are presented as numbers with percentages. Abbreviations: CT, computed tomography; DIC, disseminated intravascular coagulation; EGD, esophagogastroduodenoscopy; ETT + MV, endotracheal tube with mechanical ventilation; GI, gastrointestinal; ICU, intensive care unit. ^1^ Survival is expressed as the rate at the endpoint of the follow-up period (March 2021). The *p*-values were calculated using log-rank tests.

**Table 3:** Logistic regression models for patients in group C2 (requiring operation) and group C3 (not requiring operation).

Covariate	Univariate		Multivariate	
	Unadjusted OR (95% CI) for Requiring Operation	*p*-Value	Adjusted OR (95% CI) for Requiring Operation	*p*-Value
Age (year)	0.960 (0.919–1.003)	0.069	0.970 (0.923–1.020)	0.236
EGD grade ^1^	8.556 (1.622–45.136)	0.011	8.555 (1.559–46.942)	0.013
CT grade ^1^	2.250 (0.497–10.178)	0.292	0.350 (0.028–4.360)	0.415

^1^ The most severe grade among the esophagus, stomach, and duodenum under EGD/CT. Abbreviations: CI, confidence interval; CT, computed tomography; EGD, esophagogastroduodenoscopy; OR, odds ratio.

## Data Availability

The datasets generated and analyzed during the current study are available from the corresponding author upon reasonable request.
